# Neural and behavioral evidence for oxytocin’s facilitatory effects on learning in volatile and stable environments

**DOI:** 10.1038/s42003-024-05792-8

**Published:** 2024-01-19

**Authors:** Menghan Zhou, Siyu Zhu, Ting Xu, Jiayuan Wang, Qian Zhuang, Yuan Zhang, Benjamin Becker, Keith M. Kendrick, Shuxia Yao

**Affiliations:** 1 The Center of Psychosomatic Medicine, Sichuan Provincial Center for Mental Health, Sichuan Provincial People’s Hospital, University of Electronic Science and Technology of China, Chengdu, 611731 China; 2 The MOE Key Laboratory for Neuroinformation, School of Life Science and Technology, University of Electronic Science and Technology of China, Chengdu, China; 3 School of Sport Training, Chengdu Sport University, Chengdu, 610041 Sichuan China; 4 Center for Cognition and Brain Disorders, The Affiliated Hospital of Hangzhou Normal University, Hangzhou, Zhejiang Province China; 5 The State Key Laboratory of Brain and Cognitive Sciences, The University of Hong Kong, Hong Kong, Pokfulam China; 6 Department of Psychology, The University of Hong Kong, Hong Kong, Pokfulam China

**Keywords:** Cognitive neuroscience, Human behaviour

## Abstract

Outcomes of past decisions profoundly shape our behavior. However, choice-outcome associations can become volatile and adaption to such changes is of importance. The present study combines pharmaco-electroencephalography with computational modeling to examine whether intranasal oxytocin can modulate reinforcement learning under a volatile vs. a stable association. Results show that oxytocin increases choice accuracy independent of learning context, which is paralleled by a larger N2pc and a smaller P300. Model-based analyses reveal that while oxytocin promotes learning by accelerating value update of outcomes in the volatile context, in the stable context it does so by improving choice consistency. These findings suggest that oxytocin’s facilitatory effects on learning may be exerted via improving early attentional selection and late neural processing efficiency, although at the computational level oxytocin’s actions are highly adaptive between learning contexts. Our findings provide proof of concept for oxytocin’s therapeutic potential in mental disorders with adaptive learning dysfunction.

## Abstract (web summary)

A pharmaco-electroencephalography study shows a general facilitatory effect of intranasal oxytocin on learning under both the volatile and stable associations via improving early attentional selection and late neural processing efficiency.

## Introduction

In real life, decision-making occurs frequently every day from trifles such as when to drink a cup of coffee or go to the park to momentous decisions such as whether to quit a job or end a relationship. Outcomes of past decisions can profoundly shape our behavior, with rewarding outcomes (e.g., food or money) being reinforced and aversive ones (e.g., punishment or hurt) being avoided, as suggested by principles of reinforcement learning theory^1^. However, in a rapidly changing world associations between choices and outcomes are never invariant with choices associated with rewards in the past potentially becoming associated with punishment and vice versa. Therefore, individuals have to adapt to such changes flexibly and update their beliefs about established associations based on the difference between anticipated and actual outcomes, termed the prediction error, to optimize their decision making. However, there is evidence showing that high anxious individuals have learning deficits when choice-outcome associations are volatile^2^. Excessive fear responses have also been reported during fear extinction when conditioned stimuli are no longer paired with aversive outcomes in anxiety patients with panic or post-traumatic stress disorders^3–5^. Understanding the mechanisms underlying how these learning processes occur and whether they can be modulated is thus of importance.

One promising approach for modulating reinforcement learning is the hypothalamic neuropeptide oxytocin (OT), which plays a crucial role in modulating social behaviors and emotional processing in both animals and humans^6–8^. Given widely distributed OT receptors in the learning neural circuit^9,10^, OT can exert its effects on learning via binding to them. More specifically, intranasally administered OT has been found to facilitate learning performance with social feedback in a category association task in both Caucasian and Chinese subjects^11,12^. This enhancement effect of OT is associated with emotional, salience, and reward processing networks^11^ and may be particularly amygdala-dependent^12^. Recently using a probabilistic learning task, Zhuang et al. (2021)^13^ has also reported that intranasal OT facilitates learning by rendering the evaluation of positive (a smiley emoticon face) and negative feedback (a grumpy emoticon face) more equivalent, as reflected at a neural level in decreased feedback-related negativity (FRN) amplitude following OT relative to placebo (PLC) treatment^13^. The FRN is considered to be a reliable event-related potential (ERP) reflecting feedback evaluation with a more negative amplitude in response to negative compared to positive feedback during reinforcement learning^14,15^. These facilitatory effects of OT were maintained in a post-learning test without feedback and were also associated with attenuated error-related negativity (ERN)^13^. The ERN is associated with incorrect responses and reflects the processing of conflict monitoring at an early stage^16–18^. Similar facilitatory effects of OT on learning have also been found in high-functioning autistic adults^19^. Of note, these studies have investigated the effects of OT on reinforcement learning in stable choice-outcome associations. However, choice-outcome associations can be volatile between choices and outcomes in our rapidly changing world. Adaption to such changes are crucial for individuals to optimize decisions and can be dysfunctional in anxiety disorders^3–5^. It is therefore of importance to investigate whether OT also has modulatory effects on reinforcement learning in a volatile context and whether it does so via similar or different mechanisms to its effects in a stable context.

Furthermore, there is also a lack of evidence for behavioral computational mechanisms underlying OT’s modulatory effects on learning. Previous studies using the reinforcement learning model have shown that healthy individuals are highly adaptive in learning choice-outcome associations even in a volatile context via accelerating belief updates of choice-outcome associations and giving more weight to more recent outcomes, as reflected by higher learning rate under volatile than stable associations^2,20^. The learning rate interacts with prediction error and determines the extent to which the action value is updated during learning^1^ and has been found to be encoded in the anterior cingulate cortex^2,20^. In contrast to a faster update of action values under volatile choice-outcome associations, it is beneficial to keep choices more consistent under stable choice-outcome associations, which is evidenced by the choice consistency parameter (i.e., inverse temperature) in the reinforcement learning model^21^. These computational modeling parameters enable us to depict behavioral mechanisms underlying learning in a more elaborate way and thus may provide new possibilities for uncovering OT’s effects on reinforcement learning via a perspective of behavioral computational modeling.

**Fig. 1:**
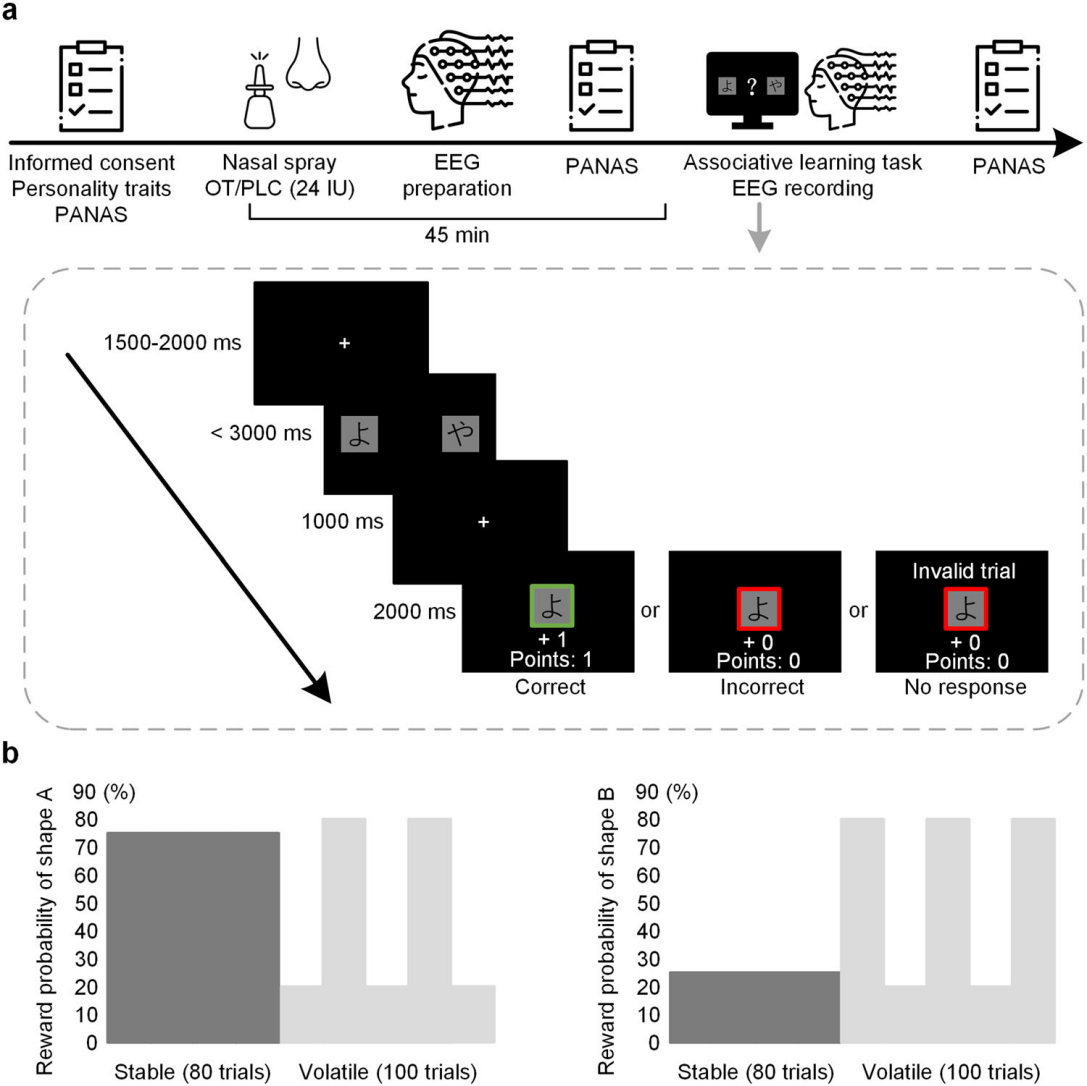
Experimental protocol and the modified associative learning task. **a** Subjects firstly filled personality trait questionnaires and then self-administered either OT (24 IU) or PLC nasal spray randomly. The associative learning task began 45 min after treatment. To further control for a potentially confounding impact of mood changes, subjects were asked to complete the Positive and Negative Affect Schedule (PANAS) 3 times (pre-treatment, pre-task and post-task). In the task, subjects were instructed to choose one of the two hiragana syllables that they considered being more likely associated with a reward based on feedback displays (i.e., ‘correct’ or ‘incorrect’ or ‘no response’). The more points they accumulated, the more payment they would obtain. **b** The associative learning task consisted of two blocks. In the stable block, choice-outcome contingencies were stable (shape A was associated with a high reward probability of 75% and shape B was associated with a low reward probability of 25%). In the volatile block, choice-outcome contingencies were volatile by switching contingencies every 20 trials (shape A) was associated with a high reward probability of 80% and shape B was associated with a low reward probability of 20% for 20 trials and vice versa in another 20 trials. There were 180 trials in total with 80 trials in the stable block and 100 trials in the volatile block. License: Icons in 1a were obtained from Flaticon.com under the free license with attribution.

Against these backgrounds, the present study combined a pharmacological challenge of intranasal OT (24 IU) in a modified associative learning task with a classical Bayesian learning model to compare OT’s effects on reinforcement learning in a volatile relative to a stable context (see Fig. 1). We also recorded subjects’ electroencephalographic signals and based on ERP indices used in the field and their functional relevance^13,22,23^, the ERN, FRN, and P300 were used as primary neural indices. The ERN and FRN are two classical ERP components associated with signaling of the mesolimbic dopamine system^24,25^ and have been widely used in previous reinforcement learning studies^13,23,26^. Attention is one of the most important factors influencing learning performance^27–29^ and effects of OT on attentional processing have also been reported in previous studies^30–32^. In the current study we therefore used the P300 associated with attentional resource allocation and stimulus salience as a primary neural index of attentional processing^33^. We also additionally measured the N2pc, an early component associated with visual selective attention^34–36^, as our secondary index of attentional processing. Thus, we had attentional ERP indices at both an early and a late stage during learning. Furthermore, given that the present study mainly focused on the effects of OT on volatile learning per se, we deliberately utilized non-social rather than social feedback to avoid observed effects being driven by social context or social salience^30,37,38^. Based on reported facilitatory effects of OT on reinforcement learning^11–13^, we hypothesized that, on the behavioral level, OT would induce similar facilitatory effects on general learning performance (e.g., choice accuracy) between stable and volatile contexts. However, behavioral computational mechanisms underlying stable and volatile contexts are different with higher choice consistency being beneficial to learning performance in the stable context but faster belief updates of choice-outcome associations being preferred in the volatile context^2,20^. The facilitatory effect of OT on learning performance of these two contexts was therefore predicted to be via different computational mechanisms (e.g., learning rate and choice consistency). On the neural level, based on previous findings that OT decreased the FRN and ERN by rendering the evaluation of positive and negative feedback more equivalent^13^ and OT’s facilitatory effects on attentional processing^30,31,39,40^, we hypothesized that while OT would decrease ERN and FRN amplitudes indicative of a diminished difference in conflict detection and evaluation between positive and negative feedback, particularly in the stable context, it would also increase N2pc and P300 amplitudes associated with visual attentional processing and resource allocation.

The present study demonstrated a general facilitatory effect of OT on increasing human learning performance independent of learning contexts, which was paralleled by a larger early event-related potential (N2pc) and a smaller late one (P300) on the neural level following OT treatment. Further computational modeling analyses revealed that while OT promoted learning by accelerating value update of outcomes in the volatile context, it did so by increasing choice consistency in the stable one. Thus the facilitatory effect of OT on learning may be exerted via improving early attentional selection and late neural processing efficiency, although at the computational level its actions are highly adaptive depending on learning contexts. Our findings provide new insights into the complexity of human learning and proof of concept for intranasal OT’s therapeutic potential in normalizing adaptive learning dysfunction.

## Results

### Demographics and questionnaires

Independent *t*-tests on personality traits (Table S1) and pre-and post-treatment measures of positive and negative mood (Table S2) revealed no significant differences between OT and PLC groups.

### Intranasal OT increases choice accuracy independent of learning context

**Fig. 2:**
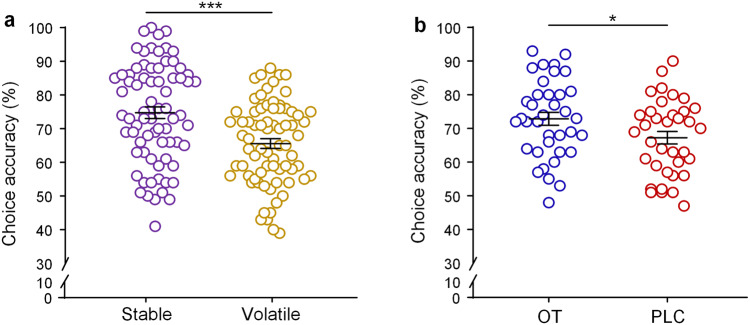
Behavioral effects of OT on choice accuracy. **a** Accuracy of choosing the optimal shape in the stable and volatile contexts across treatment groups. **b** Accuracy of choosing the optimal shape in the OT and PLC groups across contexts (*p < 0.05, **p < 0.01, ***p < 0.001). Error bars indicate standard error of the mean.

A repeated-measures ANOVA on choice accuracy of selecting the shape associated with the high-reward probability with treatment (OT vs. PLC) as between-subject factor and context (stable vs. volatile) as within-subject factor revealed a significant main effect of context (*F* (1,71) = 32.54, *p* < 0.001, ƞ_p_^2^ = 0.31; Fig. 2a), with higher choice accuracy in the stable than in the volatile context. The main effect of treatment was also significant (*F* (1,71) = 4.30, *p* = 0.042, ƞ_p_^2^ = 0.06; Fig. 2b), as reflected by a higher accuracy of subjects in the OT than in the PLC group. However, the interaction between treatment and context was not significant (*F* (1,71) = 0.10, *p* = 0.753, ƞ_p_^2^ = 0.001).

### Intranasal OT improves learning rates in the volatile context but choice consistency in the stable context

**Fig. 3:**
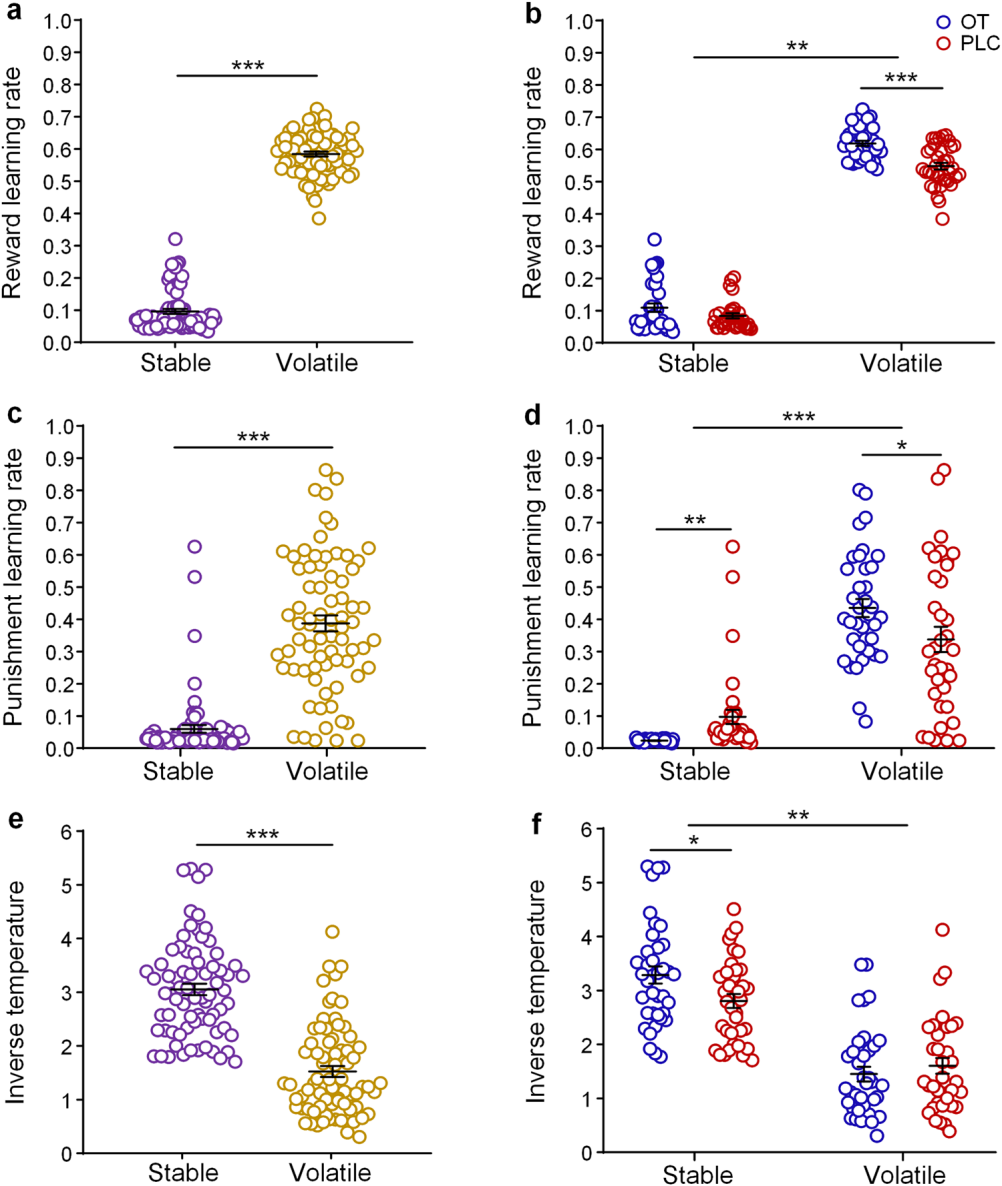
OT’s effects on reward, punishment learning rates and choice consistency as indicated by inverse temperature. **a** Reward learning rate in the stable and volatile contexts across treatment groups. **b** Reward learning rate of OT and PLC groups in the two contexts. **c** Punishment learning rate in the stable and volatile contexts across treatment groups. **d** Punishment learning rate of OT and PLC groups in the two contexts. **e** Inverse temperature in the stable and volatile contexts across treatment groups. **f** Inverse temperature of OT and PLC groups in the two association contexts (*p < 0.05, **p < 0.01, ***p < 0.001). Error bars indicate standard error of the mean.

Repeated-measures ANOVAs were employed to analyze behavioral parameters from the Reward-Punishment (RP) model. In terms of reward learning rate, a significant main effect of context (*F* (1,71) = 3504.04, *p* < 0.001, ƞ_p_^2^ = 0.98; Fig. 3a) was found, with a higher reward learning rate in the volatile than in the stable contexts. There was also a significant main effect of treatment (*F* (1,71) = 16.73, *p* < 0.001, ƞ_p_^2^ = 0.19), with reward learning rate being higher in the OT than in the PLC group. Importantly, the interaction between treatment and context was significant (*F* (1,71) = 7.72, *p* = 0.007, ƞ_p_^2^ = 0.10; Fig. 3b). Post-hoc analyses showed that while OT increased reward learning rate in the volatile context compared to PLC (*p* < 0.001), it had no significant effect in the stable context (*p* = 0.105).

For punishment learning rate, the main effect of context was significant (*F* (1,71) = 234.96, *p* < 0.001, ƞ_p_^2^ = 0.77; Fig. 3c), with punishment learning rate being higher in the volatile relative to the stable contexts. Although the main effect of treatment was not significant (*F* (1,71) = 0.15, *p* = 0.696, ƞ_p_^2^ = 0.002), the interaction between treatment and context was significant (*F* (1,71) = 16.10, *p* < 0.001, ƞ_p_^2^ = 0.19; Fig. 3d). Post-hoc tests showed that, relative to PLC, OT significantly increased punishment learning rate in the volatile (*p* = 0.046) but decreased it in the stable context (*p* = 0.001).

The repeated-measures ANOVA on inverse temperature showed a significant main effect of context (*F* (1,71) = 289.07, *p* < 0.001, ƞ_p_^2^ = 0.80; Fig. 3e), with inverse temperature being higher in the stable than in the volatile contexts. Importantly, the interaction between treatment and context was also significant (*F* (1,71) = 12.64, *p* = 0.001, ƞ_p_^2^ = 0.15; Fig. 3f). Post-hoc analyses found that OT significantly increased subjects’ choice consistency in the stable (*p* = 0.021) but not in the volatile contexts (*p* = 0.451) compared to PLC. The main effect of treatment was not significant (*F* (1,71) = 0.83, *p* = 0.364, ƞ_p_^2^ = 0.01).

### Intranasal OT has no impact on conflict detection and feedback evaluation

**Fig. 4:**
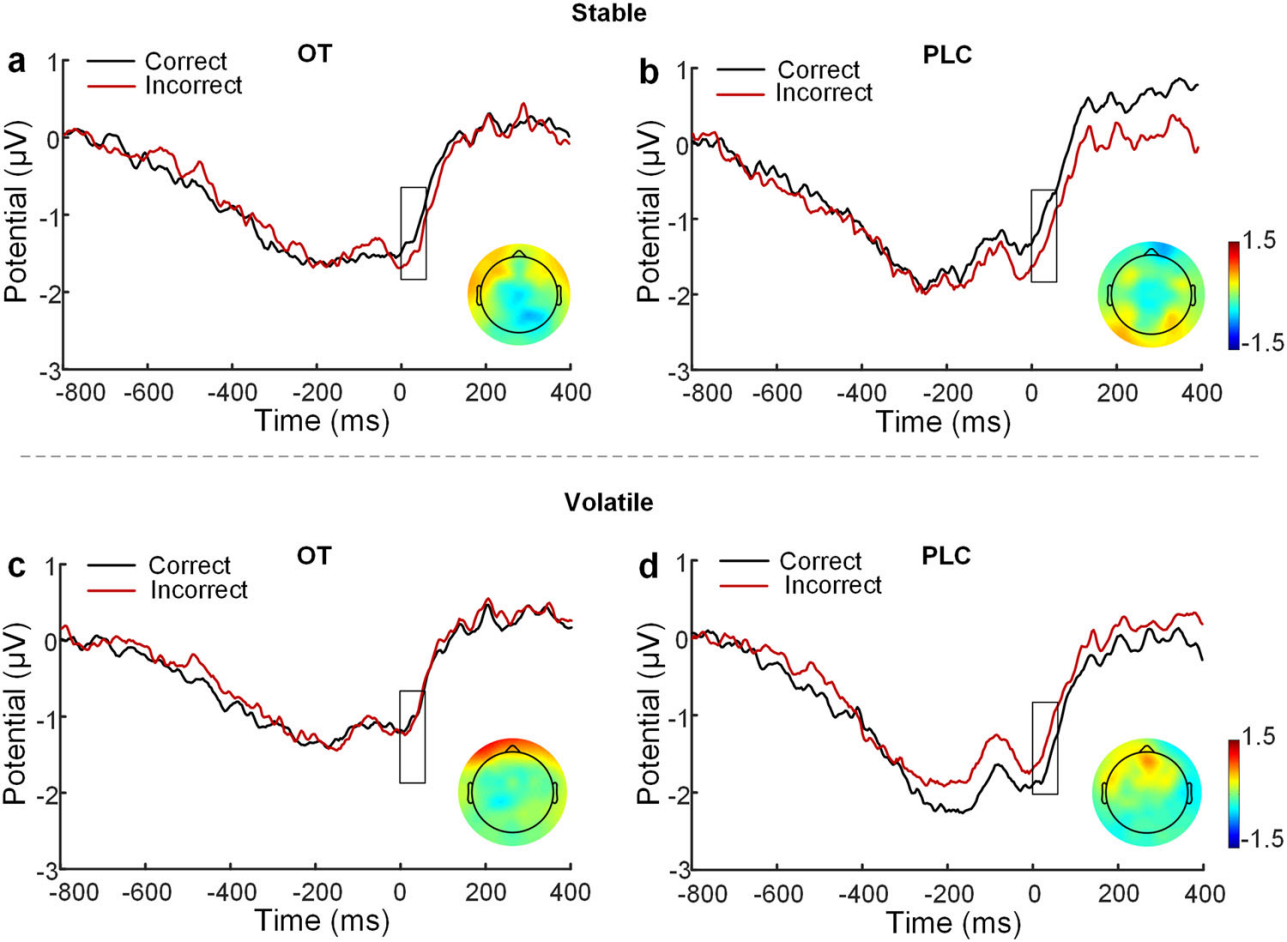
The error-related negativity (ERN) elicited in correct and incorrect trials following OT and PLC treatments. The ERN components at the electrode of FCz in correct and incorrect trials following OT and PLC treatments in the stable (**a**, **b**) and volatile (**c**, **d**) contexts respectively and topographical maps of difference waveforms.

**Fig. 5:**
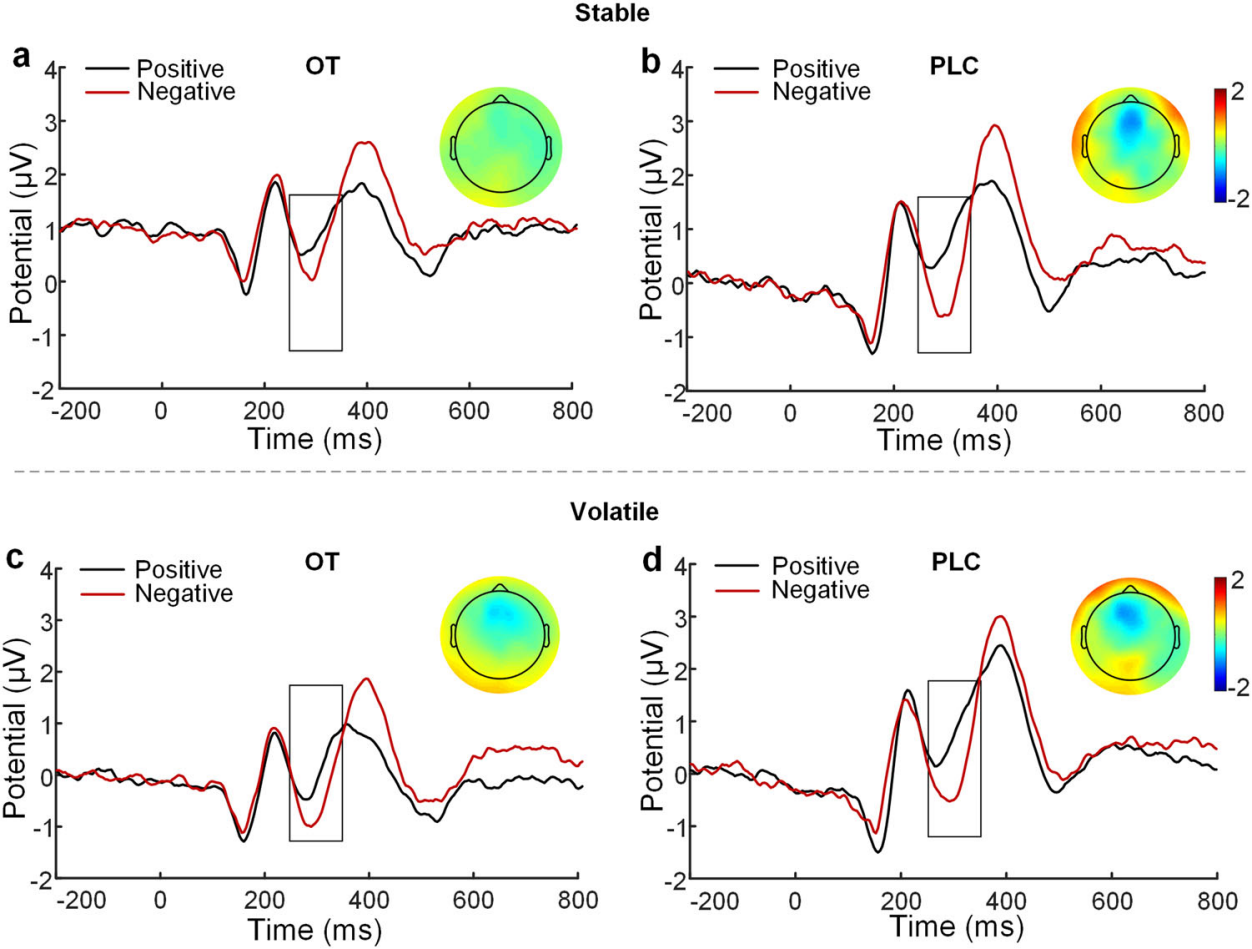
The feedback-related negativity (FRN) elicited by positive vs. negative feedback. The FRN potential at the electrode of FCz in response to positive and negative feedback following OT and PLC treatments in the stable (**a**, **b**) and volatile (**c**, **d**) contexts respectively and topographical maps of difference waveforms.

The ERN and FRN components were used to investigate whether OT’s effects on learning were exerted via modulation of conflict detection and feedback evaluation on the neural level. Results showed no significant treatment effects for either the ERN (Fig. 4) and FRN (Fig. 5) components. Given that there was an error positivity (Pe) component following the FRN, we also conducted an ANOVA on extracted Pe amplitudes and found no significant effects (all *ps* ≥ 0.210; details see Supplementary Results).

### Intranasal OT enhances neural processing efficiency and early attentional selection in both stable and volatile contexts

**Fig. 6:**
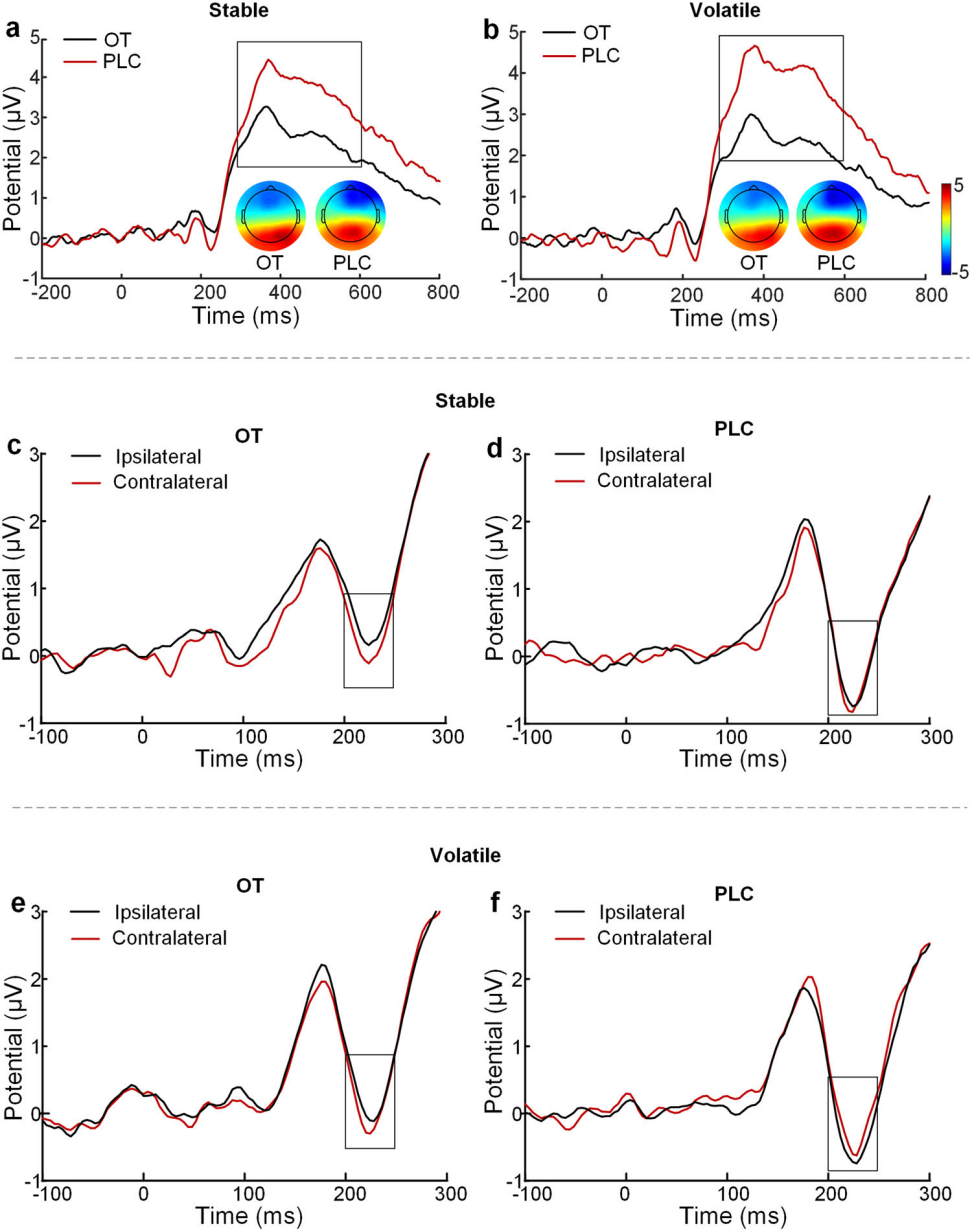
The modulatory effect of intranasal OT on the P300 and N2pc components. P300 amplitudes at the electrode Pz and topographical maps following OT and PLC treatments in the stable (**a**) and volatile (**b**) contexts. N2pc component following OT and PLC treatments in the stable (**c**, **d**) and volatile (**e**, **f**) contexts respectively.

To examine whether OT’s effects on learning were derived from evaluation of stimuli at a late stage, a repeated-measures ANOVA on the peak value of the P300 component was conducted. Results only revealed a significant main effect of treatment (*F* (1,71) = 6.88, *p* = 0.011, ƞ_p_^2^ = 0.09), with P300 amplitude being lower following OT compared with PLC treatment (3.75 ± 2.59 μv vs. 5.57 ± 3.31 μv; Fig. 6a, b). Both the main effect of context (*F* (1,71) = 0.78, *p* = 0.380, ƞ_p_^2^ = 0.01) and the interaction between treatment and context were not significant (*F* (1,71) = 3.51, *p* = 0.065, ƞ_p_^2^ = 0.05).

For the N2pc, a repeated-measures ANOVA on difference waveforms (contralateral minus ipsilateral waveform) showed a significant main effect of treatment (*F* (1,71) = 9.75, *p* = 0.003, ƞ_p_^2^ = 0.12), with a larger N2pc following OT compared with PLC treatment across contexts (–0.29 ± 0.59 vs. 0.18 ± 0.70 μv; Fig. 6c–f). However, the main effect of context (*F* (1,71) = 1.48, *p* = 0.228, ƞ_p_^2^ = 0.02) and the interaction between treatment and context were not significant (*F* (1,71) = 0.13, *p* = 0.720, ƞ_p_^2^ = 0.002). Patterns of the N2pc component provided support for preferential attentional selection of the optimal target at an early stage.

### Associations between trait anxiety, behavior, and ERP components

**Fig. 7:**
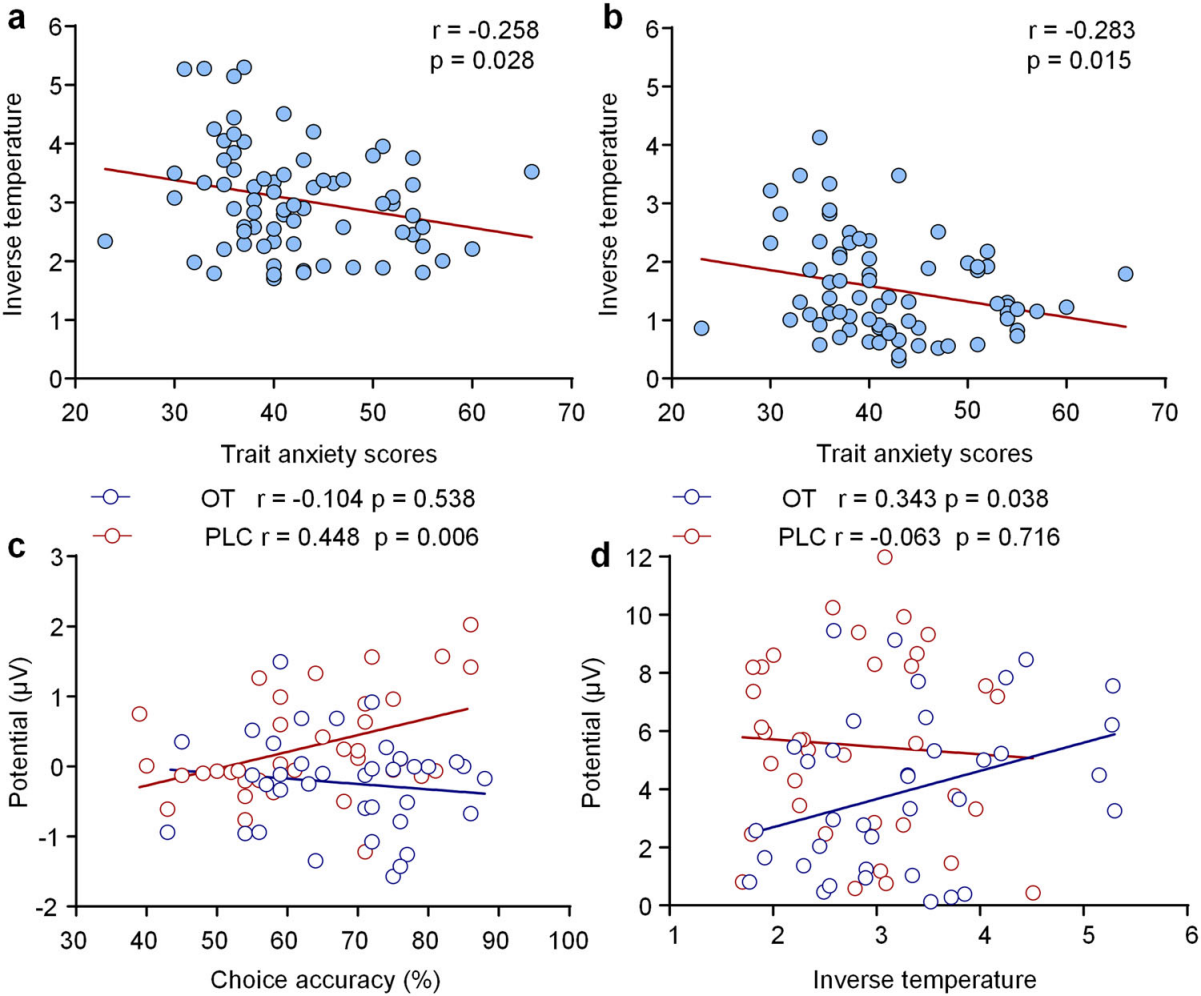
Correlations between trait anxiety, behavior, and ERP components. Negative correlations between trait anxiety scores and inverse temperature in the stable context (**a**) and in the volatile context (**b**) across groups. **c** The positive correlation between N2pc and choice accuracy following PLC but not OT treatments in the volatile context. **d** The positive correlation between P300 and inverse temperature following OT but not PLC treatments in the stable context.

Spearman correlation analyses found significant negative correlations between trait anxiety and inverse temperature in both stable (r = −0.258, *p* = 0.028; Fig. 7a) and volatile contexts (r = −0.283, *p* = 0.015; Fig. 7b) across groups, suggesting that individuals with higher trait anxiety levels exhibited less choice consistency in both contexts.

For associations between behavioral indices and ERP components that were modulated by treatment, Spearman correlation analyses showed a significant positive correlation between choice accuracy and N2pc amplitudes in the PLC (r = 0.448, *p* = 0.006) but not in the OT group (r = −0.104, *p* = 0.538; Fig. 7c) in the volatile context, indicating that higher choice accuracy was associated with a lower N2pc in the PLC group. The correlation difference was also significant between the two groups (Fisher z-score = −2.40, *p* = 0.016). However, there were no significant correlations between choice accuracy and P300 amplitudes in the stable and volatile contexts for either the OT or PLC groups (all ps ≥ 0.347). In addition, Pearson correlation analyses showed a significant positive correlation between P300 amplitudes and inverse temperature in the OT (r = 0.343, *p* = 0.038) but not the PLC group (r = −0.063, *p* = 0.716; Fig. 7d) in the stable context. However, the correlation difference was only marginal between the two groups (Fisher z-score = −1.72, *p* = 0.085).

## Discussion

The present neuropharmacological study used a modified associative learning task combined with computational modeling and ERP to investigate whether effects of intranasal OT on reinforcement learning varied as a function of different learning contexts (stable vs. volatile associations). Results showed that, compared to PLC, OT generally enhanced subjects’ choice accuracy for the optimal shape independent of learning contexts. However, further analyses based on computational modeling suggested that OT acted differently in facilitating learning in these two contexts. On the neural level, OT increased amplitudes of the N2pc but decreased amplitudes of the P300 components independent of learning contexts. In addition, we found that reinforcement learning performance was associated with individual differences in trait anxiety.

More specifically, on the behavioral level we found that choice accuracy was higher in the stable than the volatile contexts, which is predictable given that it is easier for subjects to learn stable choice-outcome associations than volatile ones. More importantly, we found that OT relative to PLC improved choice accuracy across contexts, indicating a similar enhancement effect of OT on reinforcement learning between the stable and volatile contexts. This facilitatory effect of OT is consistent with previous studies using a category association task^11,12^ or probabilistic learning task^13^, although none of these studies included manipulations of associations in a volatile context. Therefore, the present study extends previous studies by demonstrating that intranasal OT can facilitate reinforcement learning performance in humans in volatile as well as stable contexts.

Furthermore, the RP model analyses provided us more insights into the behavioral mechanisms underlying how learning occurs in stable and volatile associations. In accordance with previous studies^2,20^, reward and punishment learning rates were higher in the volatile than in the stable context. By contrast, choice consistency was higher in the stable than in the volatile context. These findings are in accordance with the reinforcement learning model such that while subjects have to update their expected values more frequently and in a timely way in the volatile environment, keeping choices more consistent is more beneficial when choice-outcome associations are stable^21^. Interestingly, significant negative correlations were found between trait anxiety scores and inverse temperature in both stable and volatile contexts, namely individuals with higher trait anxiety were more vulnerable to temporal feedback and made choices more randomly. High trait anxiety individuals also have difficulty in learning associations in an aversive environment (shock punishment), as reflected by a negative correlation between trait anxiety scores and learning rate^2^.

More importantly, OT increased both the reward and punishment learning rates in the volatile context, indicating that it accelerates expected value updates for both the positive and negative feedback. However, OT decreased punishment learning rate but enhanced inverse temperature in the stable context, suggestive of an inhibitory effect of OT on the impact from recent negative feedback but a facilitatory effect on increasing choice consistency of the optimal shape. Thus, OT’s actions on facilitating learning are highly adaptive depending on types of choice-outcome associations although via different mechanisms in stable and volatile contexts. Interestingly, while one recent behavioral study with only stable association learning reported that intranasal OT attenuated self-oriented relative to prosocial learning partially by decreasing choice consistency^41^, another one using a similar paradigm found no significant modulatory effects of a low dose (9 IU) of OT administered via a nebulizer on learning rate or choice consistency for both self-oriented and prosocial learning^42^. Thus, the effects of OT on learning may vary depending on learning orientation, doses and association contexts.

At the neural level, in contrast to our hypotheses we did not find a modulatory effect of OT on either the ERN or FRN, suggesting that the facilitatory effect of OT on reinforcement learning under stable and volatile environment is not closely associated with modulation of conflict monitoring and outcome evaluation at an early stage. However, OT has been found to decrease the FRN and ERN by rendering the evaluation of positive and negative feedback more equivalent in a probabilistic learning task^13^. The discrepancy between the findings of this previous study and the present one may be due to the different learning environments in the two studies and the types of feedback. While choice-outcome associations were stable and the task was split into a learning and a test phase in Zhuang et al. (2021)^13^, the present study included both stable and volatile associations in a learning phase and subjects were instructed to adjust their choices based on real-time feedback. Furthermore, in the Zhuang et al. (2021)^13^ social feedback was given in the form of happy and grumpy faced emoticons whereas in the current study points converted to a non-social monetary reward was used. There is growing evidence showing that OT can also have effects in nonsocial contexts^43,44^ and thus the present study also provides new support for this in reinforcement learning under volatile associations. The functional effects of OT on human behavior have consistently been demonstrated to be influenced by the nature of experimental tasks, contexts, and individual differences^6,45^.

On the other hand, OT was found to decrease the P300 amplitude in response to pairs of shapes across learning contexts. Given that the P300 has been proposed as a neural index of attentional resource allocation^33^ or neural processing efficiency at a late stage^46,47^, a smaller P300 following OT treatment may suggest that it promotes less consumption of attentional resources or more efficient neural processing of stimulus pairs. This argument can be further supported by a previous study where a reduced P300 was found to be associated with a long-term habituation effect that subjects gradually executed less cognitive control on stimulus processing over the time-course of learning^48,49^. However, such an OT-induced enhancement effect on neural processing efficiency at a late stage has to be underpinned by preferential attentional selection of the optimal target at an early stage. In other words, OT should firstly facilitate attentional selection of the optimal shape after acquiring choice-outcome associations and consequently less deep processing of stimulus pairs is required at a late stage. To validate this assumption, we further analyzed the N2pc component reflecting early visually attentional selection^34,35^. Results supported our assumption by demonstrating that OT increased the N2pc amplitude in response to the optimal shape across learning contexts, indicating that OT promoted selective attention to the optimal shape at an early stage. Similar effects of OT on improving attention to task-relevant social cues have also been reported in previous studies^50,51^. Interestingly, there was a positive correlation in the stable context between P300 amplitudes and inverse temperature in the OT group, namely subjects who kept their choices more consistently exhibited a larger P300. Thus, although OT decreased P300 amplitudes compared with PLC, in the OT group per se increased choice consistency of the optimal shape in the stable context tended to consume more attentional resources during encoding of stimulus pairs. However, given the methodology used and a lack of correlation between P300 amplitudes and choice accuracy in the present study, we cannot uncover the specific underlying mechanism and future studies are needed. Furthermore, we also found a positive correlation in the volatile context between values of the difference of the ipsilateral subtracted from the contralateral waveform and choice accuracy in the PLC group. Given that a contralateral waveform being more positive relative to the ipsilateral one in response to the optimal shape represents an opposite pattern of the N2pc or a component of distractor positivity (P_D_), this suggests that the suboptimal shape on the other side captured more attention or that the optimal shape was attentionally inhibited^52^. Thus, the positive correlation may be interpreted as subjects in the PLC group with a higher choice accuracy preferring to switch choices earlier from the current optimal shape to the suboptimal one, or to suppress the attentional processing of the current optimal shape, which would both result in better performance in the volatile context. However, OT disassociated this positive correlation seen in the PLC group, perhaps by selectively promoting choice accuracy of the optimal shape. Taken together, these findings suggest that at the neural level OT may facilitate reinforcement learning in the two learning contexts similarly by improving attentional selection of optimal shapes at an early stage and efficiency of neural processing at a late stage.

There are several limitations in the present study. First, only male subjects were recruited and thus the present findings cannot be extended to females. Secondly, although the use of non-social feedback in the present study enables us to exclude confounding effects from OT’s actions on social contexts, it is unclear whether there will be similar or distinct effects of OT on reinforcement learning using social feedback under stable and volatile associations. Future studies are needed to explore these aspects.

In summary, the present study has provided evidence for a similar facilitatory effect of OT on learning under volatile and stable choice-outcome associations using multi-methodological approaches. OT generally increased choice accuracy across the volatile and stable learning contexts, possibly via improving early attentional selection of optimal targets and efficiency of neural processing at a late stage. The computational modeling analysis further revealed that the general enhancement effect of OT on learning may be exerted via distinct behavioral mechanisms such that while it facilitated learning via accelerating the update of outcome predictions in the volatile context, it did this by improving choice consistency in the stable context. Thus, OT’s actions on facilitating learning are highly adaptive depending on types of choice-outcome associations. Findings in the present study not only provide new insights into the complexity of human learning and its modulation but also provide proof of concept evidence for the therapeutic potential of intranasal OT in mental disorders with learning dysfunction such as anxiety.

## Methods

### Participants and treatment

Eighty healthy male students (mean age = 20.65 years, SD = 1.77) were recruited from the University of Electronic Science and Technology of China (UESTC) to participate in the present double-blind, placebo-controlled, between-subject pharmacological study. Based on an a priori power analysis using the G*Power v.3.1 toolbox^53^ for a two-way mixed analysis of variance (ANOVA), the sample size was adequate to achieve a power >0.8 (effect size = 0.25, α = 0.05). All subjects self-reported being free from current or past psychiatric, neurological, or other medical conditions. They were instructed to abstain from alcohol and caffeine for the 24 h prior to the experiment and not to consume any food for 2 h before it. 7 subjects were excluded because of excessive eye movement (resulted in <50% of the trials being left for analyses in each condition; 3 subjects), self-reported fatigue (2 subjects) or noise disturbance (2 subjects) during EEG acquisition. Consequently 37 subjects in the OT group and 36 subjects in the PLC group were included in the final data analyses (mean age = 20.66 years, SD = 1.80).

To control for potential confounding effects of individual differences on personality traits and cognitive flexibility, subjects completed validated Chinese versions of psychometric questionnaires before treatment, including the Autism Spectrum Quotient^54^, State-Trait Anxiety Inventory^55^, Beck Depression Inventory^56,57^, Sensitivity to Punishment and Sensitivity to Reward Questionnaire^58^, Behavioral Inhibition System and Behavioral Activation System Scale^59^, Cognitive Flexibility Inventory^60^. To further control for a potentially confounding impact of mood changes, subjects were asked to complete the Positive and Negative Affect Schedule^61^ 3 times: when they arrived for the experiment (pre-treatment), 45 min after intranasal treatment but before the task (post-treatment) and immediately after completing the task (post-task).

Subjects were randomly assigned into two groups (OT vs. PLC) and self-administered either OT (OT-spray, Sichuan Defeng Pharmaceutical Co. Ltd, China) or PLC (placebo; identical ingredients with the OT-spray but without OT, i.e., sodium chloride and glycerin) nasal spray. Following a standardized protocol for intranasal OT administration^62^, 24 international units (IU) of OT or PLC were administered with 3 puffs to each nostril. The learning task began 45 min after treatment. All subjects were provided with written informed consent before the study and all procedures conformed with the latest version of the Declaration of Helsinki and were approved by the ethical committee of UESTC. The study was also pre-registered as a clinical trial (NCT05245708).

### Experimental task

The associative learning task was modified from Browning et al. (2015)^2^ and consisted of two blocks. In the stable block, choice-outcome contingencies were stable (shape A was associated with a high reward probability of 75% and shape B was associated with a low reward probability of 25%). In the volatile block, choice-outcome contingencies were volatile by switching contingencies every 20 trials (shape A was associated with a high reward probability of 80% and shape B was associated with a low reward probability of 20% for 20 trials and vice versa in another 20 trials)^2^. There were 180 trials in total with 80 trials in the stable block and 100 trials in the volatile block. Block order was counterbalanced across subjects for each treatment group.

Each trial started with a jittered fixation (1500–2000 ms) that changed to a question mark simultaneously with the presentation of a pair of Japanese hiragana syllables (“よ” and “や”). These syllables were unfamiliar to the Chinese subjects and were presented for 3000 ms or until response. Subjects were instructed to choose one of the two hiragana syllables that they considered being more likely associated with a reward. Selection of the left shape was made by pressing the “F” key and the right shape by pressing the “J” key. Associations and positions of the two shapes were counter-balanced across subjects. After a response, there was another fixation interval (1000 ms) before the presentation of feedback (2000 ms). In the feedback display, a green (correct response) or a red (incorrect or no response) frame appeared around the answer shape, with the real-time rewarding points in the current trial and cumulative scores across trials being presented below it. A correct response was rewarded by one point (“+1”) and an incorrect response was given zero points (“+0”). Given that we mainly focused on OT’s effects on dynamic learning per se, we deliberately utilized these non-social rather than social feedback to avoid observed effects being driven by social context or salience^30,37,38^. If subjects did not respond in time, a warning message “invalid trial” would be presented above the frame. There were no cues indicating the block type before each block. Subjects were instructed to optimize their choices in real-time based on the feedback information to obtain as many points as possible in order to maximize their payment. They were all clearly informed that their final payment would correspond to the total points earned during the task plus a basic participant fee. Each point was worth 15 RMB cents. Consistent with Browning et al. (2015)^2^, the two blocks were completed sequentially without breaks to avoid interruptions in the time course of association learning, and lasted approximately 20 min. Ten practice trials were performed by each subject before the main task. Subjects were asked to keep their eyes on the displayed fixation cross to minimize blinking and eye movements during the experiment.

### EEG data collection and analyses

The EEG was recorded at a sampling rate of 500 Hz using a 64-channel ActiCap system with a Quick Amp amplifier (Brain Products GmbH, Germany). Signals of all channels were online referenced to the Cz electrode (the international 10–20 system). Electrode impedances were kept below 5 kΩ. The EEGLAB 14.1.1 toolbox^63^ was used to preprocess the raw data. The EEG data were down-sampled to 250 Hz, filtered with a Hamming windowed sinc FIR filter separately for high- and low-pass filters (high-pass: 0.1 Hz, −6 dB cutoff: 0.05 Hz; low-pass: 40 Hz, −6 dB cutoff: 45 Hz), and offline re-referenced to the average reference. Correction of eye movement artifacts was conducted by independent component analysis (ICA).

For the ERN, in accordance with previous studies^13^, an epoch from 800 ms before and 500 ms after the response was extracted with the time window pre-response from 800 ms to 700 ms serving as the ERN baseline. The FRN was time-locked to 200 ms pre-feedback and 1000 ms post-feedback with a baseline from 200 ms to 0 ms pre-feedback. To remove the remaining artifacts after ICA, epochs with voltage values exceeding ±80 µV were further discarded from analyses^64–66^. This resulted in an average of 7.88% of ERN trials and 6.95% of FRN trials were excluded from further analyses. The ERN was defined as the peak difference after responses between correct and incorrect trials in the time window of 0–60 ms at electrode FCz^13^. The FRN was calculated as the peak difference between positive and negative feedback in the time window of around 252–352 ms at electrode FCz^67^.

For the P300, EEG data was extracted from 200 ms before and 1000 ms after the onset of displayed stimuli from correct trials with a baseline of 200 ms to 0 ms pre-onset. Similar to ERN and FRN, epochs with voltage values exceeding ±80 µV were further discarded from analyses and an average of 5.75% of trials were excluded. The P300 peak amplitude was calculated in the time window of 300–600 ms at the electrode Pz^68,69^. The corresponding remaining epochs of each ERP component were averaged for each condition in each subject. To examine whether OT facilitated attentional selection of the optimal shape at an early stage, we further analyzed the N2pc component, which is an explicit index of early visual attentional selection with a more negative amplitude in response to contralateral relative to ipsilateral targets^34–36^. The N2pc component was segmented using a time window between 100 ms pre-stimulus and 400 ms post-stimulus from correct trials, with approximately 1.85% of trials deleted from analyses. The ipsilateral waveform was computed as the average of the left-sided electrode (PO7) to the left-sided targets and the right-sided electrode (PO8) to the right-sided targets, whereas the contralateral waveform was computed as the average of the left-sided electrode to the right-sided targets and the right-sided electrode to the left-sided targets^34,35^. Peak amplitude of N2pc was calculated in the 200–252 ms time window at PO7 and PO8 electrodes^70,71^.

### Computational model

To capture subjects’ learning performance, especially the computational basis, in a more sensitive way we employed a widely validated reinforcement learning model with a hierarchical Bayesian parameter estimation referred to as the RP model^72,73^. The RP model posits that positive and negative feedback affect perseverative learning behavior differently and can provide more elaborate parameters to depict learning behavior via trial-by-trial analyses. This model is described by the following equations:

$${v}_{A,(t+1)}=\left\{\begin{array}{c}{v}_{A,(t)}+{\alpha }^{{pos}}\times \left({R}_{(t)}-{v}_{A,(t)}\right),{to}\,{positive}\,{feedback}\\ {v}_{A,(t)}+{\alpha }^{{neg}}\times \left({R}_{(t)}-{v}_{A,(t)}\right),{to}\,{negative}\,{feedback}\end{array}\right.\,$$

and

$${v}_{-A,(t+1)}={v}_{-A,(t)}$$

*v*_*A*,(*t*+1)_ is subjects’ predicted outcome value of shape A on trial *t* + 1 and *v*_*A*,(*t*)_ is the expected value of shape A on trial *t*. *α* ^*pos*^ is the learning rate of reward and *α*^*neg*^ is the learning rate of punishment (ranging from 0 to 1). The learning rate indicates the extent to which the prediction error is utilized to update the expected value and can reflect the speed of updating and learning based on reward or punishment outcomes. *R*_(*t*)_ represents the actual outcome on trial *t* and *R*_(*t*)_ – *v*_*A,(t)*_ therefore means the prediction error on trial *t*. *v-*_*A*,(*t*+1)_ is the predicted outcome value of the unchosen option. Note that only information of the chosen stimulus is updated in this model. The probability of choosing each shape is then modeled using a softmax choice function as follows:

$${P}_{t+1}\left(A\right)=\frac{1}{{1+e}^{-\beta * \left(\right.({v}_{t+1}\left(A\right)-{v}_{t+1}(B))}}$$

*P* is the probability of choosing shape A on trial *t* + 1. The inverse temperature parameter *β* represents subjects’ choice consistency. A smaller *β* indicates more random choices, namely less consistent choice making, and vice versa. The probability of choosing shape B is 1 - *P*.

The RP model was estimated using a Markov Chain Monte Carlo algorithm implemented in the hBayesDM package in R and all Rhat values were less than 1.1^74^. Normal priors have been used for hyperparameters (μ ~ normal (0,1); σ ~ normal (0,0.2)). Based on previous learning studies involved in the volatile context^2,20,72,75^, we considered another two reinforcement learning models (Rescorla-Wagner (Delta) Model and Experience-Weighted Attraction Model), which are also based on the classic theory of Rescorla-Wagner and widely used in the field (for details of these two models see Supplementary Computational model). To determine whether the RP model best fitted our behavioral data, we compared it with these two models using the Leave-One-Out Information Criterion (LOOIC). The LOOIC estimates pointwise out-of-sample prediction accuracy from a fitted Bayesian model^76^, with a lower value of LOOIC suggesting a better model-fit. The results indicated that the RP model was the best fitting model (see Table 1).

**Table 1:** Model comparison based on LOOIC in different contexts of OT and PLC groups.

Model	Stable_OT	Stable_PLC	Volatile_OT	Volatile_PLC
Reward and Punishment Model	2663	3117	3999	4084
Rescorla-Wagner (Delta) Model	2709	3036	4010	4147
Experience-Weighted Attraction Model	2675	3099	4017	4151

To further confirm whether this model sufficiently captured subjects’ actual choice behavior, we simulated subjects’ choice of the optimal shape in each trial for each condition and found that this model fitted well with subjects’ actual choices (see Fig. S1). For validation of the winning model, subjects’ choices in each trial were simulated by using posterior prediction checks of these estimated parameters in the RP model for each condition. Results using the simulated data replicated findings of subjects’ actual choices (details see Supplementary Computational model).

### Statistics and reproducibility

Independent *t*-tests were conducted to compare group differences on questionnaire scores of mood and personality traits. For behavioral data, we focused on the choice accuracy of selecting the optimal shape. A 2 × 2 ANOVA with treatment (OT vs. PLC) as between-subject factor and context (stable vs. volatile) as within-subject factor was performed on choice accuracy. After fitting the RP model in each condition, behavioral parameters including the reward learning rate, punishment learning rate, and inverse temperature were obtained and were also analyzed using the treatment × context ANOVAs respectively.

For the ERP data, to examine whether treatment effects on reinforcement learning varied as a function of different learning contexts (stable vs. volatile associations), we performed a 2 × 2 ANOVA with treatment as between-subject factor and context as within-subject factor on ERN (difference amplitude: incorrect minus correct response), FRN (difference amplitude: negative minus positive feedback), N2pc (difference amplitude: contralateral minus ipsilateral waveform), and P300. The Greenhouse-Geisser correction was employed whereby assumptions of sphericity were violated.

Furthermore, correlations between trait anxiety, behavioral responses, modeling parameters and neural signals were tested using Spearman or Pearson correlations depending on distribution of the data. Correlation differences between treatments were tested using the Fisher z-transformation test.

### Reporting summary

Further information on research design is available in the Nature Portfolio Reporting Summary linked to this article.

### Supplementary information

Supplementary Information

Reporting summary

## Data Availability

All data that support the findings of this study are openly available via the Open Science Framework Repository (https://osf.io/5u837/).
