# Supplementary material for: Neural and behavioral evidence for oxytocin’s facilitatory effects on learning in volatile and stable environments
Source: Commun Biol. 2024 Jan 19;7:109. doi: 10.1038/s42003-024-05792-8 (PMC10799007; doi:10.1038/s42003-024-05792-8)
Supplement: Supplementary file 1 — Supplementary Information [file 42003_2024_5792_MOESM1_ESM.pdf]

SUPPLEMENTARY MATERIALS

Neural and behavioral evidence for oxytocin's facilitatory effects on learning in volatile and stable environments

Menghan Zhou, Siyu Zhu, Ting Xu, Jiayuan Wang, Qian Zhuang, Yuan Zhang, Benjamin Becker, Keith M. Kendrick, Shuxia Yao*

Supplementary results

Demographics and questionnaires

Table S1. Statistics of age and questionnaire scores of personality traits and cognitive flexibility

Characteristic	OT (M ± SD)	PLC (M ± SD)	*t*_values	*p*_values
Age	20.54±1.68	20.78±1.93	-0.561	0.576
Autism Spectrum Quotient (ASQ)	22.92±4.79	20.78±5.60	1.758	0.083
State-Trait Anxiety Inventory				
-Trait Anxiety Inventory (TAI)	41.95±7.56	42.53±8.80	-0.303	0.763
-State Anxiety Inventory (SAI)	39.30±7.24	40.92±8.91	-0.853	0.396
Beck Depression Inventory (BDI-II)	8.70±6.95	9.33±7.86	-0.363	0.717
Sensitivity to Punishment and Sensitivity to Reward Questionnaire (SPSRQ)				
-Sensitivity to Punishment (SP)	11.08±3.38	10.47±4.18	0.686	0.495
-Sensitivity to Reward (SR)	9.81±2.91	9.33±2.93	0.698	0.487
Behavioral Inhibition System and Behavioral Activation System Scale				
-Behavioral Inhibition System (BAS)	23.22±4.78	24.81±4.36	-1.483	0.142
-Behavioral Activation System (BIS)	15.11±3.24	15.56±2.61	-0.649	0.519
Cognitive Flexibility Inventory (CFI)				
-Control	24.51±3.88	23.97±4.75	0.534	0.595
-Alternatives	46.84±7.88	46.97±6.93	-0.077	0.939

Values are presented as mean ± SD. Abbreviations: OT, oxytocin; PLC, placebo.

Table S2. Statistics of the Positive and Negative Affect Schedule scores

	OT (M ± SD)	PLC (M ± SD)	*t*-values	*p*-values
Positive and Negative Affect Schedule$^{pre\text{-}treatment}$				
-Positive	26.24±6.37	26.11±6.35	0.089	0.930
-Negative	14.59±4.88	16.81±6.91	-1.583	0.118
Positive and Negative Affect Schedule$^{post\text{-}treatment}$				
-Positive	23.14±6.89	24.06±7.33	-0.553	0.582
-Negative	12.73±4.68	13.78±5.09	-0.916	0.363
Positive and Negative Affect Schedule$^{post\text{-}task}$				
-Positive	22.49±6.45	23.39±7.38	-0.557	0.580
-Negative	11.84±4.73	12.03±3.57	-0.193	0.847

Values are presented as mean ± SD. Abbreviations: OT, oxytocin; PLC, placebo.

Computational model

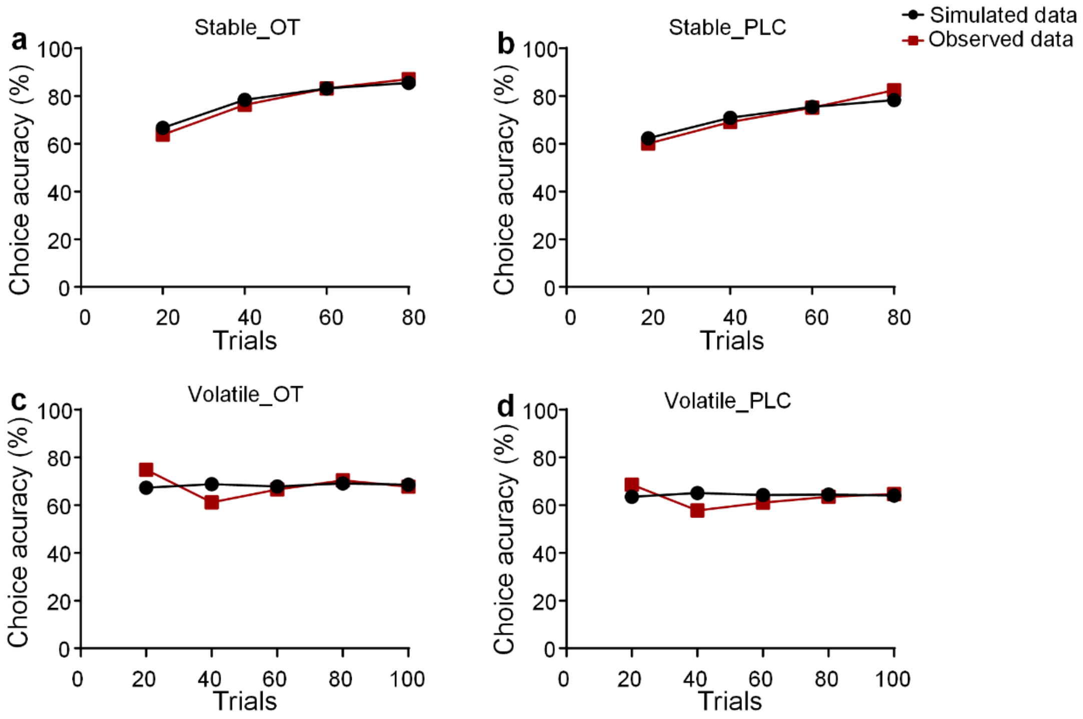

Fig. S1 Simulation of the fitted model. The averaged probability of choosing the optimal shape in the observed data was presented against the stimulated data for each condition (a-d).

Based on previous learning studies involved in the volatile context[1–4], we also considered another two reinforcement learning models that are commonly used and fit into our experimental paradigm. Similar to the Reward-Punishment (RP) model, these two models are also developed based on the classic theory of Rescorla-Wagner. One is the Rescorla-Wagner (Delta) Model[5] in which prediction error is also utilized to update the expected value. However, different from the RP model, it only has one learning rate parameter and one inverse temperature parameter that do not distinguish between reward or punishment outcomes. Rescorla-Wagner (Delta) Model is described by the following equation:

$$v_{A,(t+1)} = v_{A,t} + \alpha \times \left(R_{(t)} - v_{A,(t)}\right)$$

The meanings of these parameters are the same to those in the RP model.

The other one is the Experience-Weighted Attraction Model[3,6] which includes three parameters: the learning rate, experience decay factor and inverse temperature. In this model, unlike the RP model, it is hypothesized that individuals learn from the relative weight of past experience. This model is described by the following equations:

$$n_{A,(t+1)} = n_{A,t} \times \rho + 1,$$

and

$$v_{A,(t+1)} = (v_{A,t} \times \varphi \times n_{A,t} + \lambda_t)/n_{A,(t+1)}$$

$n_{A,(t+1)}$ is the "experience weight" of stimulus A on trial t+1, which is updated on each trial based on the experience decay factor ρ. $v_{A,(t+1)}$ is the value of choice A on trial t+1, $\lambda_t \in \{0,1\}$ is for the outcome received in response to that choice and φ is the decay factor for the previous payoffs. Learning rate in the Rescorla-Wagner (Delta) Model here is equivalent to (1 - φ).

For validation of the winning model, subjects' choices in each trial were simulated by using posterior prediction checks of these estimated parameters in the RP model for each condition. A repeated-measures ANOVA on choice accuracy of the optimal shape based on the simulated data revealed a significant main effect of context (F (1,71) = 47.13, $p < 0.001$, $\eta_p^2 = 0.40$) and treatment (F (1,71) = 6.43, $p = 0.013$, $\eta_p^2 = 0.08$). However, the interaction between context and treatment was not significant (F (1,71) = 1.05, $p = 0.310$, $\eta_p^2 = 0.02$). These results were similar to the pattern as found in the observed data, suggesting that the fitted RP model was effective in capturing the observed data.

Intranasal OT has no impact on conflict detection and feedback evaluation in both stable and volatile contexts

The ERN and FRN components were used to investigate whether OT's faciliatory effect on learning exerted via modulation of conflict detection and feedback evaluation on the neural level. For ERN, a repeated-measures ANOVA on difference waveforms (incorrect minus correct response) showed no significant main effect of context ($F\,(1,71) = 2.58$, $p = 0.113$, $\eta_p^2 = 0.03$) or treatment ($F\,(1,71) = 0.22$, $p = 0.642$, $\eta_p^2 = 0.003$). The interaction between treatment and context was also not significant ($F\,(1,71) = 0.95$, $p = 0.332$, $\eta_p^2 = 0.01$) (Fig. 4). With respect to the FRN reflecting feedback evaluation, a repeated-measures ANOVA on difference waveforms (negative minus positive feedback) showed no significant main effect of context ($F\,(1,71) = 0.002$, $p = 0.962$, $\eta_p^2 = 0.001$) or treatment ($F\,(1,71) = 0.90$, $p = 0.345$, $\eta_p^2 = 0.01$) or interaction between treatment and context ($F\,(1,71) = 1.58$, $p = 0.213$, $\eta_p^2 = 0.02$) (Fig. 5). Given that there was an error positivity (Pe) component following the FRN, we also conducted an ANOVA on difference waveforms and found no significant main effects for either context ($F\,(1,71) = 1.603$, $p = 0.210$) or treatment ($F\,(1,71) = 0.024$, $p = 0.876$). The interaction between treatment and context was also not significant ($\mathrm{F}\,(1,71) = 0.879$, $p = 0.352$). The absence of treatment effects on the ERN and FRN demonstrated that the enhancement effect of OT on learning was not associated with conflict detection and feedback evaluation.

Reference

1. Browning, M., Behrens, T. E., Jocham, G., O'reilly, J. X. & Bishop, S. J. Anxious individuals have difficulty learning the causal statistics of aversive environments. *Nat Neurosci* **18**, 590–596 (2015).
2. Pulcu, E. *et al.* The Effects of the Angiotensin II Receptor Antagonist Losartan on Appetitive Versus Aversive Learning: A Randomized Controlled Trial. *Biol Psychiatry* **86**, 397–404 (2019).
3. Den Ouden, H. E. M. *et al.* Dissociable effects of dopamine and serotonin on reversal learning. *Neuron* **80**, 1090–1100 (2013).
4. Behrens, T. E. J., Woolrich, M. W., Walton, M. E. & Rushworth, M. F. S. Learning the value of information in an uncertain world. *Nat Neurosci* **10**, 1214–1221 (2007).
5. Zhang, L., Lengersdorff, L., Mikus, N., Gläscher, J. & Lamm, C. Using reinforcement learning models in social neuroscience: frameworks, pitfalls and suggestions of best practices. *Soc Cogn Affect Neurosci* **15**, 695–707 (2020).
6. Camerer, C. & Ho, T.-H. Experience-Weighted Attraction Learning in Coordination Games: Probability Rules, Heterogeneity, and Time-Variation. *J Math Psychol* **42**, 305–326 (1998).
